# Novel Design of Iridium Phosphors with Pyridinylphosphinate Ligands for High-Efficiency Blue Organic Light-emitting Diodes

**DOI:** 10.1038/srep38478

**Published:** 2016-12-08

**Authors:** Zheng-Guang Wu, Yi-Ming Jing, Guang-Zhao Lu, Jie Zhou, You-Xuan Zheng, Liang Zhou, Yi Wang, Yi Pan

**Affiliations:** 1 State Key Laboratory of Coordination Chemistry, Collaborative Innovation Center of Advanced Microstructures, School of Chemistry and Chemical Engineering, Nanjing University, Nanjing 210093, P. R. China; 2 State Key Laboratory of Rare Earth Resource Utilization, Changchun Institute of Applied Chemistry, Chinese Academy of Sciences, Changchun 130022, P. R. China

## Abstract

Due to the high quantum efficiency and wide scope of emission colors, iridium (Ir) (III) complexes have been widely applied as guest materials for OLEDs (organic light-emitting diodes). Contrary to well-developed Ir(III)-based red and green phosphorescent complexes, the efficient blue emitters are rare reported. Like the development of the LED, the absence of efficient and stable blue materials hinders the widely practical application of the OLEDs. Inspired by this, we designed two novel ancillary ligands of phenyl(pyridin-2-yl)phosphinate (ppp) and dipyridinylphosphinate (dpp) for efficient blue phosphorescent iridium complexes (dfppy)_2_Ir(ppp) and (dfppy)_2_Ir(dpp) (dfppy = 2-(2,4-difluorophenyl)pyridine) with good electron transport property. The devices using the new iridium phosphors display excellent electroluminescence (EL) performances with a peak current efficiency of 58.78 cd/A, a maximum external quantum efficiency of 28.3%, a peak power efficiency of 52.74 lm/W and negligible efficiency roll-off ratios. The results demonstrated that iridium complexes with pyridinylphosphinate ligands are potential blue phosphorescent materials for OLEDs.

In recent years, considerable attention has been attached to organic light emitting diodes (OLEDs) due to their successful applications in solid-state lighting and full-color flat-panel display. To the present, cyclometalated iridium (Ir) (III) complexes are still the most promising phosphorescent guest materials for highly efficient OLEDs because of their short excited lifetime (microsecond time-scale), color tuning flexibility, high quantum yields and thermal stability^1^,^2^,^3^,^4^,^5^,^6^,^7^,^8^,^9^,^10^,^11^,^12^,^13^,^14^. Like light-emitting diode (LED), blue light is indispensable among the trichromatic emissions, which plays a pivotal role in saving energy and achieving pure white emission with high color rendering index (CRI). In comparison with extensively studied iridium-based red and green phosphorescent complexes, the efficient blue emitters are limited and the performances of blue OLEDs are still not satisfactory. Therefore, achieving stable and highly efficient blue phosphorescent iridium complexes and their corresponding devices still remains a significant challenge.

It is well known that functionalized 2-phenylpyridine derivatives were most often used for blue Ir(III) emitters as the main ligands. Generally, the modification of pyridine unit with electron-donating group would heighten the LUMO level, while the decoration of phenyl unit with electron-drawing group can lower the HOMO level. In addition, it is also possible to enlarge the HOMO–LUMO energy gap by designing and employing other *N*-heterocyclic moieties to replace the pyridyl or phenyl moiety of phenylpyridine ligand^15^,^16^,^17^,^18^,^19^,^20^,^21^,^22^,^23^. Furthermore, another way is using ancillary ligands with strong coordination fields, which also can simplify the synthetic conditions. Up-to-date, most efficient blue Ir(III) emitters were designed by combining these strategies, such as FIrpic (Ir(III)bis(4,6-(difluorophenyl)pyridinato-*N,C*’)picolinate)^24^,^25^, and FIr6 (Ir(III)bis(4,6-(difluorophenyl) pyridinato-*N,C*^2’^)-tetrakis(1-pyrazolyl)borate)^26^,^27^, etc.^28^,^29^,^30^,^31^,^32^,^33^,^34^. Among them, FIrpic is the most widely used blue emitter due to its good device performances, simple molecular structure and ease of synthesis. Although tremendous efforts have been made to develop appropriate host materials and optimize device structures, the efficiency and stability of OLEDs based on FIrpic are still suffering from acid-induced decomposition and poor electron mobility^35^. It is generally known that charge balance is vital for high efficiency and low efficiency roll-off of OLEDs. In most OLEDs, the electron mobility of the electron transport material is always lower than the hole mobility of the hole transport material. Therefore, bipolar host materials and phosphorescent emitters with high electron mobility were considered to be helpful to balance the distribution of carries^36^,^37^,^38^,^39^,^40^,^41^,^42^,^43^. Several groups have attempted to design highly efficient blue Ir(III) complexes with high electron mobility containing appropriate ligands^44^,^45^,^46^,^47^,^48^,^49^,^50^,^51^. For example, the Chi group used pyrazole and triazole because these nitrogen heterocycles owning more negative framework of ligands will increase the electron affinity and may be beneficial in improving the electron mobility^18^,^19^.

In this context, we designed two novel ancillary ligands of phenyl(pyridin-2-yl)phosphinate (ppp) and dipyridinylphosphinate (dpp) for blue bis-cyclometalated Ir(III) complexes (dfppy)_2_Ir(ppp) and (dfppy)_2_Ir(dpp) (dfppy = 2-(2,4-difluorophenyl)pyridine, Fig. 1). The introduction of phosphoryl (P = O)moiety will improve the coordination fields of the ancillary ligand to make a hypsochromic shift. More importantly, P = O is a strong electron-withdrawing group capable of polarizing the molecule, which has been widely used in bipolar host materials and electron transport materials based on its excellent electron transport property^52^,^53^,^54^,^55^,^56^,^57^,^58^,^59^,^60^,^61^. In our former publications, we also reported some efficient OLEDs with Ir(III) complexes containing the electron transport P = O moiety^38^,^62^,^63^. The devices based on (dfppy)_2_Ir(ppp) and (dfppy)_2_Ir(dpp) displayed prominent electroluminescence (EL) performances with a peak current efficiency of 58.78 cd/A, a maximum external quantum efficiency (*EQE*_max_) near 30% and low efficiency roll-off due to the introduction of P = O moiety and nitrogen heterocycle which can effectively balance the injection and transportation of charges and confine excitons in the emissive layer.

Figure 1 showed the synthesis procedure for (dfppy)_2_Ir(ppp) and (dfppy)_2_Ir(dpp) complexes. The reaction of 2-bromopyridine with *n*-butyllithium and dichlorophenylphosphine (or phosphorus trichloride) gave the dipyridinyl phenyl phosphine (or tripyridinyl phosphine), which were further reacted with hydrogen peroxide and sodium hydroxide through a sequential oxidation and alkaline hydrolysis process to afford the corresponding Na(ppp) and Na(dpp) salts. Then, the two pyridinylphosphinates were employed with [(dfppy)_2_Ir(*μ*-Cl)]_2_ dimer to form the relevant (dfppy)_2_Ir(ppp) and (dfppy)_2_Ir(dpp) complexes, which were characterized in detail by ^1^H NMR, ^31^P NMR, MS, and HRMS; single-crystal structure of (dfppy)_2_Ir(ppp) was also proved by X-ray crystallography (Fig. 2(a)). The Ir metal is coordinated by two C^N main ligands and one phenyl(pyridin-2-yl)phosphinate ancillary ligand. The coordination ligands around the iridium center are in an octahedral geometry with the *cis*-N,O, *cis*-C,C, and *trans*-N,N chelating atoms. The Ir-C, Ir-N and Ir-O bond lengths are in the ranges of 1.965(8)–2.019(8) Å, 2.007(8)–2.176(7) Å and 2.169(6) Å, respectively, within the normal values for cyclometalated Ir(III) complexes (Table S1, Supporting Information). Furthermore, both complexes show good thermal stability, evaluated by thermogravimetric analysis (TGA) under a nitrogen atmosphere. The decomposed temperatures (T_d_) of 5% weight loss are 389 °C for (dfppy)_2_Ir(ppp) and 396 °C for (dfppy)_2_Ir(dpp), respectively (Fig. S1), which are much higher than that of FIrpic (T_d_ = 344 °C)^64^. These results suggest that both Ir(III) complexes are suitable for the application in OLEDs to guarantee no any decomposition during a long operation time.

The ultraviolet-visible (UV-vis) absorption and photoluminescence (PL) spectra of (dfppy)_2_Ir(ppp) and (dfppy)_2_Ir(dpp) (CH_2_Cl_2_ solution, 10^−5^ M) at RT are depicted in Fig. 2(b), and the relevant data are collected in Table 1. In the absorption spectra the two complexes show intense bands before 350 nm due to the spin-allowed ligand-to-ligand charge transfer (^1^LLCT) transitions of cyclometalated and ancillary ligands. While the metal-to-ligand charge transfer (^1^MLCT and ^3^MLCT) absorptions are distinguished at approximately 380 nm with lower extinction coefficients, which were confirmed by the theoretical calculation (Fig. S2 and Table S2). Both complexes show intense blue phosphorescence with maximum emission peaks at 471 nm for (dfppy)_2_Ir(ppp) with a shoulder emission at 497 nm and 470 nm for (dfppy)_2_Ir(dpp) with a weaker emission at 496 nm, respectively. To ensure the reliability of quantum efficiency, the integrating-sphere system was used for measuring the absolute photoluminescence quantum yields in deaerated CH_2_Cl_2_, and the results were 0.35 for (dfppy)_2_Ir(ppp) and 0.25 for (dfppy)_2_Ir(dpp), respectively. In addition, the lifetimes are in the range of microseconds for the two novel complexes (1.44 μs for (dfppy)_2_Ir(ppp) and 1.34 μs for (dfppy)_2_Ir(dpp), respectively) (Fig. S3). The short lifetimes would improve the spin-state mixing and suppress the excitons annihilation.

The HOMO/LUMO energy levels of the iridium complexes are closely related to the choice of charge transport and host materials as well as design of OLED structure. Therefore, cyclic voltammetry experiments were carried out to calculate the HOMO and LUMO levels of the complexes (Fig. S4). The HOMO levels of (dfppy)_2_Ir(ppp) and (dfppy)_2_Ir(dpp) were found to be −5.95 and −5.96 eV, respectively, while the LUMO levels of (dfppy)_2_Ir(ppp) and (dfppy)_2_Ir(dpp) were estimated as −3.00 and −2.95 eV from the HOMO levels and UV-vis absorption spectra (Table S3).

To evaluate the electroluminescence (EL) performances of (dfppy)_2_Ir(ppp) and (dfppy)_2_Ir(dpp), they were used as the dopants in OLEDs with the configuration of indium tin oxide (ITO)/MoO_3_ (3 nm)/TAPC (di-[4-(*N,N*-ditolyl-amino)-phenyl]cyclohexane) (50 nm)/(dfppy)_2_Ir(ppp) or (dfppy)_2_Ir(dpp) (*x* wt%): 26DCzPPy (2,6-bis(3-(carbazol-9-yl)phenyl)pyridine) (15 nm)/TmPyPB (1,3,5-tri(*m*-pyrid-3-yl-phenyl)benzene) (50 nm)/LiF (1 nm)/Al (100 nm). MoO_3_ and LiF served as hole- and electron-injecting interface modified materials, respectively. TAPC owning high hole mobility (1 × 10^−2^ cm^2^/V · s) and high-lying LUMO level (−1.8 eV) was used as hole transport/electron block layer (HTL/EBL)^65^, while TmPyPB with high electron mobility (1 × 10^−3^ cm^2^/V · s) and low-lying HOMO level (−6.7 eV) was used as electron transport/hole block layer (ETL/HBL)^66^. Bipolar material 26DCzPPy was chosen as host because it nearly possesses the equal electron mobility (μ_e_) and hole mobility (μ_h_) values of 1 × 10^−5^ − 8 × 10^−5^ cm^2^/V · s at an electric field between 6.0 × 10^5^ and 1.0 × 10^6^ V/cm which benefit the electron – hole balance in emissive layer^39^. Figure 3 shows the chemical structures of the used materials as well as the energy level diagram of the devices. Apparently, the HOMO/LUMO levels of (dfppy)_2_Ir(ppp) and (dfppy)_2_Ir(dpp) are within those of 26DCzPPy. Therefore, carriers are expected to transport easily between layers, and excellent carrier trapping would be the main mechanism in these devices. More importantly, carriers (hole and electron) will be well confined within the emissive layer, and the triplet excitons quenching will be effectively avoided. By optimizing the dopant concentrations (Table S4 and Fig. S5), two devices based on (dfppy)_2_Ir(ppp) (PPP-1) or (dfppy)_2_Ir(dpp) (DPP-1) with 10 wt% concentration showed the highest efficiencies. So far FIrpic is considered to be one of the best blue phosphorescent guest materials, devices with FIrpic dopant with above device structure (pic-1) were also fabricated to compare with our reported results based on (dfppy)_2_Ir(ppp) and (dfppy)_2_Ir(dpp), and the highest performances were obtained on the doping concentration of 16 wt% (Fig. S6).

The EL spectra, current density – voltage – luminance (*J* – *V* – *L*), current efficiency - luminance (*η*_*c*_ - *L*) and power efficiency - luminance (*η*_p_ - *L*) curves of the devices (PPP-1 and DPP-1) are shown in Fig. 4 (the curves for pic-1 are listed in Fig. S6), and the device performance data are listed in Table 2. Both devices showed typical emission maxima at 475 nm with shoulders at 500 nm, in accordance with the PL of the Ir(III) complexes in CH_2_Cl_2_ solutions. The CIE (Commission Internationale de L’Eclairage) color coordinates are (*x* = 0.13, *y* = 0.37) for PPP-1 and DPP-1 operated at 10 mA cm^−2^, corresponding to the sky-blue region. The absence of residual emission from the host suggests the complete energy transfer from 26DCzPPy to emitters. Both PPP-1 and DPP-1 devices displayed good EL performances with the maximum luminance above 20000 cd/m^2^, the peak current efficiency (*η*_c,max_) over 50 cd/A and the peak power efficiency (*η*_p,max_) over 50 lm/W. Respectively, device PPP-1 gave a maximum luminance of 32923 cd/m^2^ at a driving voltage of 8.9 V, a *η*_c,max_ of 52.49 cd/A, a *EQE*_max_ of 25.2% and a *η*_p,max_ of 51.50 lm W^−1^. Device DPP-1 exhibited a little higher EL efficiencies with a *η*_c,max_ of 55.79 cd A^−1^, a EQE_max_ of 26.4% and a *η*_p,max_ of 50.56 lm/W. Noticeably, with gradual enhancement of the current density, both devices have small efficiency roll-off ratios. For device PPP-1, the *η*_c_ at practical brightness of 100 cd m^−2^ and 1000 cd/m^2^ are 49.71 and 46.81 cd/A, respectively. For DPP-1, these values are still high as 52.79 and 48.06 cd/A, respectively. These performances are higher than those of the FIrpic-based device (pic-1), which showed a *η*_c,max_ of 35.73 cd/A and a *η*_p,max_ of 23.37 lm/W using same materials and device structure (Table 2). Their outstanding EL performances may be attributed to the following facts: the LUMO/HOMO levels of the iridium complexes situated within those of 26DCzPPy, so the carriers can be trapped almost simultaneously by the phosphorescent dopants when they are injected into emission layer, which will benefit for the improvement of exciton recombination efficiency. The electron transport group P=O and nitrogen-containing heterocycle in the iridium complexes as well as lower LUMO level will be helpful for the electron transport to improve hole - electron balance. A better balanced charge transport will promote the recombination of electrons and holes and broaden the recombination zone as well as lead to the suppressed current leakage in the devices. As a result, a slow decay of the device efficiencies with increasing driving voltage demonstrates their relatively high device stability^67^,^68^.

In order to further improve the EL performances, another hole transport material TcTa (4,4’,4”-tris(carbazol-9-yl)triphenylamine) was introduced as the “hole ladder” layer due to its matched HOMO level (5.80 eV) between TAPC and 26DCzPPy (Fig. 3). The devices with the configuration of ITO/MoO_3_ (3 nm)/TAPC (50 nm)/TcTa (5 nm)/(dfppy)_2_Ir(ppp) or (dfppy)_2_Ir(dpp) (10 wt%): 26DCzPPy (15 nm)/TmPyPB (50 nm)/LiF (1 nm)/Al (100 nm) were named as PPP-2 and DPP-2, respectively. The EL characteristics of these devices are exhibited in Fig. 5, and the key EL values are also collected in Table 2.

It is observed that the performances of (dfppy)_2_Ir(ppp)-based device (PPP-2) are not markedly improved with a *η*_c,max_ of 45.71 cd/A and a *η*_p,max_ of 37.78 lm/W, respectively. However, the device based on (dfppy)_2_Ir(dpp) (DPP-2) achieved better EL performances with a lower turn-on voltage of 3.2 V, a higher *η*_c,max_ of 58.78 cd/A, a *EQE*_max_ of 28.3% and a *η*_p,max_ of 52.74 lm/W, respectively. Most importantly, with the current density increase, the efficiency roll-off values are very small. For example, even at the luminance of 1000 cd/m^2^, current efficiency as high as 48.79 cd/A can be retained by the device DPP-2 with *EQE* near 25%. To the best of our knowledge, such performances are amongst the best results achieved from blue phosphorescent biscyclometalated Ir(III) emitters^35^,^69^. This ascendant result is attributed to more balanced electrons and holes transportation and distribution within emissive layer. For PPP-2, because the addition of TcTa will facilitate the hole injection into emitting layer, and more holes accumulation would lower the device efficiency consequently. But for device DPP-2, the pyridinyl and P=O groups in (dfppy)_2_Ir(dpp) are helpful for electron transport, which causes the higher electron density within the emitting layer of DPP-2; therefore, the insertion of TcTa layer assists to balance the carriers and improve the EL efficiency. The electron mobility test results of the Ir(III) complexes (Fig. 6) would confirm this hypothesis.

The TEL (transient electroluminescence) experiments with the strucutre of ITO/TAPC (50 nm)/Ir(III) complex (60 nm)/LiF (1 nm)/Al (100 nm) were conducted (Fig. S7). Here, TAPC functions as hole- transport layer, while Ir(III) compounds function as both emissive and electron-transport layer. The experimental results showed that the electron mobilities of (dfppy)_2_Ir(ppp), (dfppy)_2_Ir(dpp) and FIrpic are between 6.77–6.92 × 10^−6^, 7.50–7.84 × 10^−6^ and 4.60–5.14 × 10^−6^ cm^2^/V · s, respectively, under an electric field range from 1150 (V/cm)^1/2^ to 1300 (V/cm)^1/2^ (Fig. 6). The results suggest that the electron mobilities of both (dfppy)_2_Ir(dpp) and (dfppy)_2_Ir(ppp) are better than that of FIrpic, and the data of (dfppy)_2_Ir(dpp) is a little higher than that of (dfppy)_2_Ir(ppp). The excellent electron mobility of (dfppy)_2_Ir(ppp) and (dfppy)_2_Ir(dpp) would promote the electrons injection and transport, leading to the balanced hole-electrons distribution, broaden exciton recombination zone, and suppressed current leakage. Consequently, the annihilation and dissociation of excitons at high current density would be inhibited effectively^70^,^71^, which contributes to improved recombination probability, high device efficiency and slight efficiency roll-off.

In conclusion, two novel bis-cyclometalated Ir(III) complexes containing phenyl(pyridin-2-yl)phosphinate (ppp) or dipyridinylphosphinate (dpp) as the ancillary ligands were synthesized and applied for blue phosphorescent OLEDs. The related photophysical, electrochemical and electroluminescent properties were thoroughly investigated. Device PPP-1 based on (dfppy)_2_Ir(ppp) with the structure of ITO/MoO_3_ (3 nm)/TAPC (50 nm)/(dfppy)_2_Ir(ppp) (10 wt%):26DCzPPy (15 nm)/TmPyPB (50 nm)/LiF (1 nm)/Al (100 nm) displayed good EL performances with a turn-on voltage of 2.9 V, a *η*_c,max_ of 52.49 cd/A, a *EQE*_max_ of 25.2%, a *η*_p,max_ of 51.50 lm/W with low efficiency roll-off. The (dfppy)_2_Ir(dpp)-based device DPP-2 with the configuration of ITO/MoO_3_ (3 nm)/TAPC (50 nm)/TcTa (5 nm)/(dfppy)_2_Ir(dpp) (10%):26DCzPPy (15 nm)/TmPyPB (50 nm)/LiF (1 nm)/Al (100 nm) exhibited excellent EL performances of a *η*_c,max_ of 58.78 cd/A, a *EQE*_max_ of 28.3%, a *η*_p,max_ of 52.74 lm/W with small efficiency roll-off ratios. The prominent performances of (dfppy)_2_Ir(ppp) and (dfppy)_2_Ir(dpp)-based devices maybe due to the suitable HOMO/LUMO levels and high electron mobility, which are helpful in balancing the injection, transport and distribution of carriers. The results demonstrated that these iridium complexes with pyridinylphosphinate ancillary ligands are potential blue phosphorescent materials for facilitating the commercial applications of OLEDs.

## Methods

### Materials and measurements

All reagents and chemicals were purchased from commercial sources and used without further purification. ^1^H and ^31^P NMR spectra were measured on a Bruker AM 400 spectrometer. Mass spectra (MS) were obtained with ESI-MS (LCQ Fleet, Thermo Fisher Scientific). High resolution mass spectra (HRMS) were measured with a LTQ-Orbitrap XL (Thermofisher, USA). Absorption and photoluminescence spectra were measured on a UV-3100 spectrophotometer and a Hitachi F-4600 photoluminescence spectrophotometer, respectively. The decay lifetimes and absolute photoluminescent quantum yields were measured with an Edinburgh Instruments FLS-920 fluorescence spectrometer equipped with an integrating sphere in degassed CH_2_Cl_2_ solution at room temperature. Cyclic voltammetry measurements were conducted on a MPI-A multifunctional electrochemical and chemiluminescent system (Xi’an Remex Analytical Instrument Ltd. Co., China) at room temperature, with a polished Pt plate as the working electrode, platinum thread as the counter electrode and Ag-AgNO_3_ (0.1 M) in CH_3_CN as the reference electrode, *tetra*-n-butylammonium perchlorate (0.1 M) was used as the supporting electrolyte, using Fc^+^/Fc as the internal standard, the scan rate was 0.1 V/s.

### X-ray crystallography

The single crystals of complexes and ligand were carried out on a Bruker SMART CCD diffractometer using monochromated Mo Kα radiation (λ = 0.71073 Å) at room temperature. Cell parameters were retrieved using SMART software and refined using SAINT on all observed reflections. Data were collected using a narrow-frame method with scan widths of 0.30° in ω and an exposure time of 10 s/frame. The highly redundant data sets were reduced using SAINT and corrected for Lorentz and polarization effects. Absorption corrections were applied using SADABS supplied by Bruker. The structures were solved by direct methods and refined by full-matrix least-squares on *F*2 using the program SHELXS-97. The positions of metal atoms and their first coordination spheres were located from direct-methods E-maps; other non-hydrogen atoms were found in alternating difference Fourier syntheses and least-squares refinement cycles and, during the final cycles, refined anisotropically. Hydrogen atoms were placed in calculated position and refined as riding atoms with a uniform value of Uiso.

### Fabrication and measurements of OLEDs

Indium-tin-oxide (ITO) coated glass with a sheet resistance of 10 Ω/sq was used as the anode substrate. Prior to film deposition, patterned ITO substrates were cleaned with detergent, rinsed in de-ionized water, dried in an oven, and finally treated with oxygen plasma for 5 minutes at a pressure of 10 Pa to enhance the surface work function of ITO anode (from 4.7 to 5.1 eV). All the organic layers were deposited with the rate of 0.1 nm/s under high vacuum (≤2 × 10^−5^ Pa). The doped layers were prepared by co-evaporating dopant and host material from two individual sources, and the doping concentrations were modulated by controlling the evaporation rate of dopant. MoO_3_, LiF and Al were deposited in another vacuum chamber (≤8.0 × 10^−5^ Pa) with the rates of 0.01, 0.01 and 1 nm s^−1^, respectively, without being exposed to the atmosphere. The thicknesses of these deposited layers and the evaporation rate of individual materials were monitored in vacuum with quartz crystal monitors. A shadow mask was used to define the cathode and to make ten emitting dots (with the active area of 10 mm^2^) on each substrate. Device performances were measured by using a programmable Keithley source measurement unit (Keithley 2400 and Keithley 2000) with a silicon photodiode. The EL spectra were measured with a calibrated Hitachi F–7000 fluorescence spectrophotometer. Based on the uncorrected EL fluorescence spectra, the Commission Internationale de l’Eclairage (CIE) coordinates were calculated using the test program of Spectrascan PR650 spectrophotometer. The EQE of EL devices were calculated based on the photo energy measured by the photodiode, the EL spectrum, and the current pass through the device.

### Measurement and calculation of electron mobility

The electron mobility measurement of (dfppy)_2_Ir(ppp) and (dfppy)_2_Ir(dpp) was used the transient EL technique on the devices of ITO/TAPC (50 nm)/Ir(III) complex (60 nm)/LiF (1 nm)/Al (100 nm). A rectangular voltage pulse (amplitude 8~10 V, pulse duration 50 *μ*s) was applied to an OLED by using a pulse generator (Rigol Model DG1022). The time-dependent EL was detected by placing a miniature photomultiplier tube (PMT, Hammamatsu Model H6780) directly on the top of the OLED. The output photocurrent from the PMT was sourced into a sensing resistor in which the transient EL signal was displayed on a storage oscilloscope (Tektronix Model TDS2000B). The electron mobility can be roughly calculated with the equation of *μ*_e_ = *d*^2^/(*t*_*d*_ · *V*), where *d* is the thickness of the emitting layer, *V* is the driving voltage, and *t*_*d*_ is the delay time.

## Additional Information

**How to cite this article**: Wu, Z.-G. *et al*. Novel Design of Iridium Phosphors with Pyridinylphosphinate Ligands for High-Efficiency Blue Organic Light-emitting Diodes. *Sci. Rep.* **6**, 38478; doi: 10.1038/srep38478 (2016).

**Publisher’s note:** Springer Nature remains neutral with regard to jurisdictional claims in published maps and institutional affiliations.

## Supplementary Material

Supporting Information

## Figures and Tables

**Figure 1:**
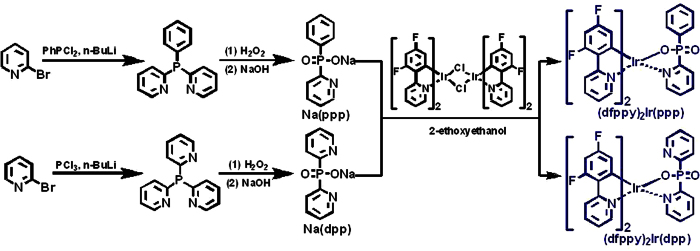
Synthesis routes for iridium complexes (dfppy)_2_Ir(ppp) and (dfppy)_2_Ir(dpp).

**Figure 2:**
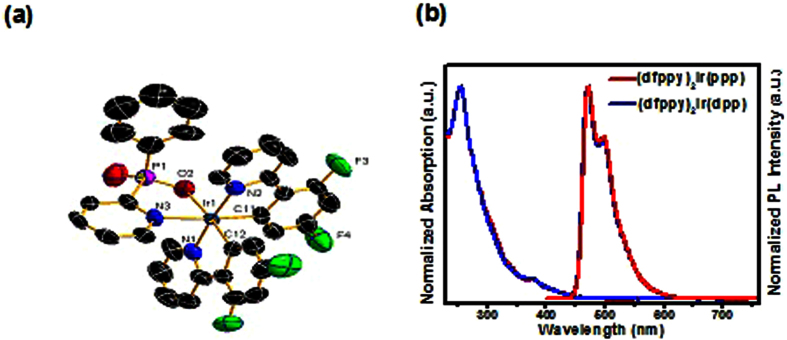
(a) Oak Ridge thermal ellipsoidal plot (ORTEP) diagrams of (dfppy)_2_Ir(ppp) with the atom-numbering Figure. Ellipsoids are drawn at 30% probability level. Crystal data (CCDC: 1439293) C_33_H_20_F_4_IrN_3_O_2_P, *M*w = 789.69; Monoclinic space group, P2_**1**_/c; *a* = 14.542(16) Å, *b* = 13.256(14) Å, *c* = 16.511(18) Å, *α* = 90°, *β* = *γ* = 99.868(18)°, *V* = 3136(6) Å^3^; *Z* = 4, *F*(000) = 1532, GOF on *F*^2^ = 1.126, *R*_1_ = 0.0633, *wR*_2_ = 0.1667. (**b**) UV-vis absorption and PL spectra of (dfppy)_2_Ir(ppp) and (dfppy)_2_Ir(dpp) in CH_2_Cl_2_ solution at room temperature (RT).

**Figure 3:**
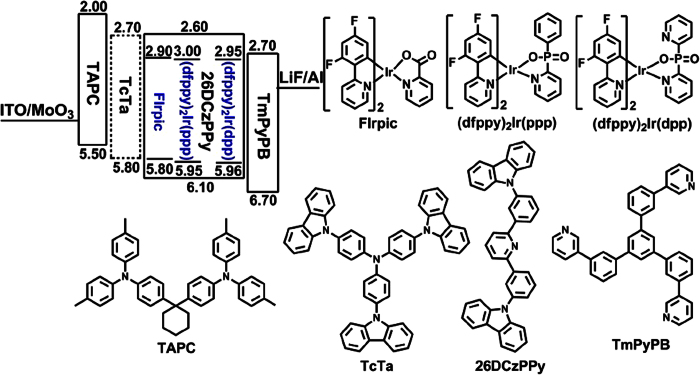
HOMO/LUMO energy level diagram of materials and their molecular structures.

**Figure 4:**
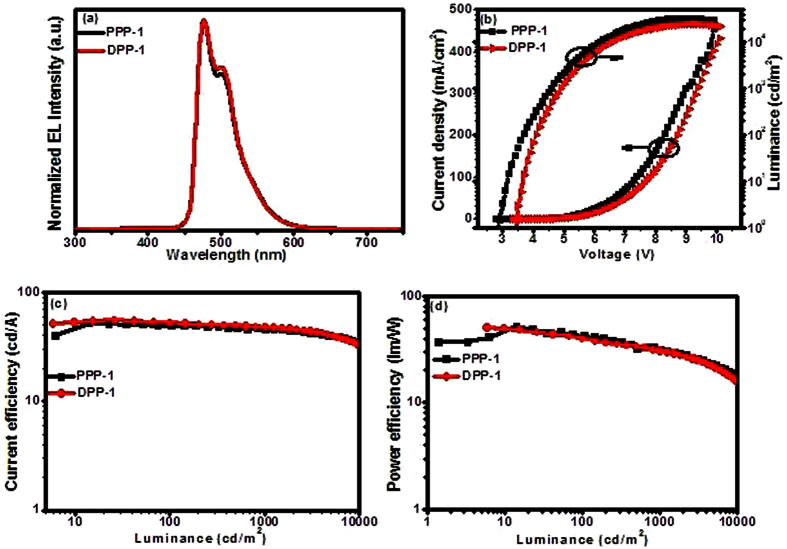
OLED performances with the structure of ITO/MoO_3_ (3 nm)/TAPC (50 nm)/(dfppy)_2_Ir(ppp) or (dfppy)_2_Ir(dpp) (10 wt%): 26DCzPPy (15 nm)/TmPyPB (50 nm)/LiF (1 nm)/Al (100 nm): (**a**) EL spectra at 10 mA; (**b**) current density - luminance – voltage curves; (**c**) current efficiency – luminance curves; (**d**) power efficiency – luminance curves.

**Figure 5:**
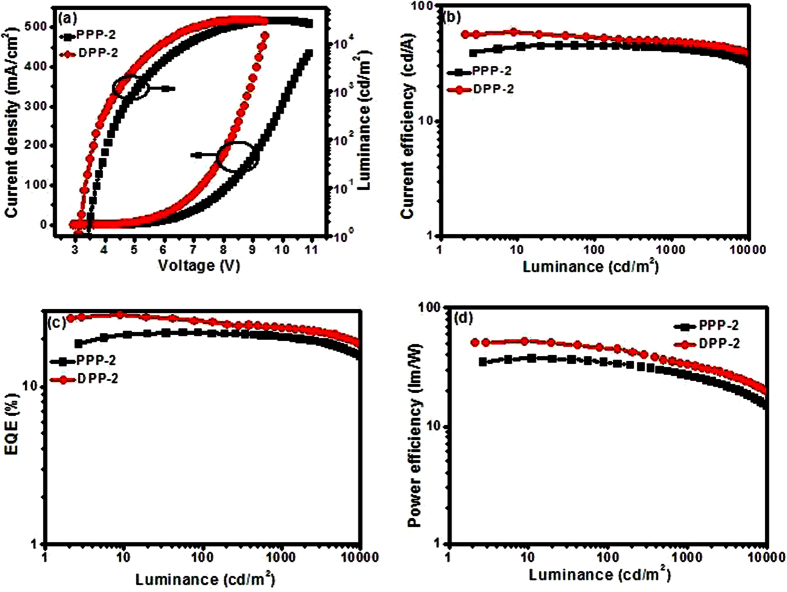
OLED performances with the configuration of ITO/MoO_3_ (3 nm)/TAPC (50 nm)/TcTa (5 nm)/(dfppy)_2_Ir(ppp) or (dfppy)_2_Ir(dpp) (10 wt%): 26DCzPPy (15 nm)/TmPyPB (50 nm)/LiF (1 nm)/Al (100 nm): (**a**) current density and luminance versus voltage; (**b**) current density - luminance – voltage curves; (**c**) current efficiency – luminance curves;” should be (**b**) current density – luminance curves; (**c**) external quantum efficiency – luminance curves; (**d**) power efficiency – luminance curves.

**Figure 6:**
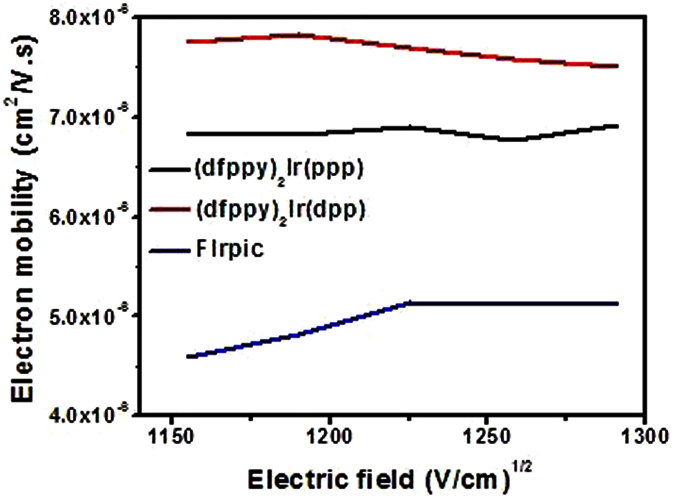
Electron mobility versus electric field in the thin films of (dfppy)_2_Ir(ppp), (dfppy)_2_Ir(dpp) and FIrpic.

**Table 1:** Physical property data of (dfppy)_2_Ir(ppp) and (dfppy)_2_Ir(dpp).

Compound	*T*_d_^a^ [°C]	**λ**_abs_ [nm]	**λ**_em_ [nm]	*Ф*^b^ [%]	*τ* ^c^ [μs]	Band gap [eV]	HOMO/LUMO^d^ [eV]
(dfppy)_2_Ir(ppp)	389	256/379	471/497	35	1.44	2.95	−5.95/−3.00
(dfppy)_2_Ir(dpp)	396	256/377	470/496	25	1.34	3.01	−5.96/−2.95

^a^*T*_d_: decomposition temperature; ^b^*Ф*: absolute photoluminescence quantum yields in deaerated CH_2_Cl_2_ with a integrating-sphere system; ^c^Measured at RT in CH_2_Cl_2_ (10^−5^ M); ^d^ HOMO/LUMO energy levels calculated using the cyclic voltammetry (CV) diagram with ferrocene as the internal standard and UV-vis spectra in CH_2_Cl_2_.

**Table 2:** Key EL data of the OLEDs.

Device	*V*_turn-on_^a^ [V]	*L*_max_(voltage)^b^ [cd/m^2^(V)]	*η*_c.max_^c^ [cd/A]	*η*_c,100/1000_^d^ [cd/A]	*η*_p.max_^e^ [lm/W]	*η*_p,100/1000_^f^ [lm/W]	*EQE*_max_^g^ [%]
PPP-1	2.9	32923(8.9)	52.49	49.71/46.81	51.50	42.19/32.66	25.2
DPP-1	3.5	24629(9.2)	55.79	52.79/48.06	50.56	40.20/30.80	26.4
Pic-1	3.2	33094(9.3)	35.73	5.45/9.95	23.37	4.18/22.41	16.9
PPP-2	3.4	30138(9.9)	45.71	45.71/43.32	37.78	35.01/27.76	21.9
DPP-2	3.2	31270(8.9)	58.78	54.24/48.79	52.74	46.34/33.31	28.3

^a^*V*_turn-on_: turn-on voltage recorded at a brightness of 1 cd/m^2^; ^b^*L*_max_: maximum luminance; ^c^*η*_c,max_: maximum current efficiency; ^d^current efficiencies measured at brightness of 100 cd/m^2^ and 1000 cd/m^2^; ^e^*η*_p,max_: maximum power efficiency; ^f^power efficiencies measured at brightness of 100 cd/m^2^ and 1000 cd/m^2^; ^g^*EQE*_max_: maximum external quantum efficiency.
