# Supplementary material for: Novel Design of Iridium Phosphors with Pyridinylphosphinate Ligands for High-Efficiency Blue Organic Light-emitting Diodes
Source: Sci Rep. 2016 Dec 8;6:38478. doi: 10.1038/srep38478 (PMC5144070; doi:10.1038/srep38478)
Supplement: Supporting Information [file srep38478-s1.pdf]

Supporting Information

Novel design of iridium phosphors with pyridinylphosphinate ligands for high-efficiency blue organic light-emitting diodes

Zheng-Guang Wu,[1] Yi-Ming Jing,[1] Guang-Zhao Lu,[1] Jie Zhou,[1] You-Xuan Zheng,[1]* Liang Zhou,[2]* Yi Wang[1]* and Yi Pan[1]

[1] State Key Laboratory of Coordination Chemistry, Collaborative Innovation Center of Advanced Microstructures, School of Chemistry and Chemical Engineering, Nanjing University, Nanjing 210093, P. R. China, *e-mail: yxzheng@nju.edu.cn, wangyi@nju.edu.cn

[2] State Key Laboratory of Rare Earth Resource Utilization, Changchun Institute of Applied Chemistry, Chinese Academy of Sciences, Changchun 130022, P. R. China, *e-mail: zhoul@ciac.ac.cn

1. Experimental section

1.1 Synthesis of $[(dfppy)_2Ir(\mu\text{-}Cl)]_2$

1.2 Synthesis of sodium phenyl(pyridin-2-yl)phosphinate (Nappp)

1.3 Synthesis of sodium dipyridinyl phosphinate (Nadpp)

1.4 Synthesis of $(dfppy)_2Ir(ppp)$

1.5 Synthesis of $(dfppy)_2Ir(dpp)$

2. Supplementary data

2.1 Crystallographic data for complex $(dfppy)_2Ir(ppp)$

2.2 Thermogravimetric analysis (TGA) of $(dfppy)_2Ir(ppp)$ and $(dfppy)_2Ir(dpp)$

2.3 The calculated and experimental measured absorption spectra of the $(dfppy)_2Ir(ppp)$ and $(dfppy)_2Ir(dpp)$ complexes.

2.4 The lifetime curves of $(dfppy)_2Ir(ppp)$ and $(dfppy)_2Ir(dpp)$

2.5 Cyclovoltammety (CV) diagrams and HOMO/LUMO levels of $(dfppy)_2Ir(ppp)$ and $(dfppy)_2Ir(dpp)$

2.6 The PL spectra of the $(dfppy)_2Ir(ppp)$ dopant in 26DCzPPy host in (a) CH_2Cl_2 solution and (b) solid film.

2.7 Optimization of the doping concentration of Ir(III) emitters

2.8 OLED performances of FIrpic device

2.9 Measurement of electron mobility

1. Experimental Section

1.1 Synthesis of [(dfppy)$_2$Ir(μ-Cl)]$_2$. $IrCl_3$ (2.30 g, 6.23 mmol) and dfppy (2.50 g, 13.08 mmol) were dissolved in 24 mL 2-$EtOCH_2CH_2OH$: H_2O (3: 1, v/v), and refluxed at 120 ℃ for 24 h. After cooling to room temperature, the yellow precipitate was filtered and washed with acetone: ethanol = 15 mL: 15 mL. The washed product was dried under vacuum with a (2.38 g, 1.96 mmol) 62.91% yield. ESI-MS: [M] calcd for $C_{44}H_{24}N_4Cl_2F_8Ir_2$, 1216.05; found [M+H] 1217.65.

1.2 Synthesis of sodium phenyl(pyridin-2-yl)phosphinate (Nappp). A solution of 2.5 N *n*-butyl-lithium (6.40 mL, 16.00 mmol) in 15 mL of Et_2O was cooled to -90 ℃ and 2-bromopyridine (2.52 g, 15.94 mmol) in Et_2O (6 mL) at -90 ℃ was added quickly and the dark red solution stirred at -90 ℃ for 4 h. A solution of dichlorophenylphosphine (1.44 g, 8.04 mmol) in Et_2O (10 mL) was added dropwise during 1 h at -90 ℃ and the solution stirred at -90 ℃ for 2 h before warming slowly to room temperature. The tan-coloured mixture was extracted with H_2SO_4 (20 mL, 2N) and the extract made alkaline with saturated NaOH solution. The solid product was collected and recrystallised from acetone-petroleum (1:1, v/v) to get pure product 0.84g with 39.59% yield. Then the product (0.84 g, 3.17 mmol) was dissolved in DMSO (5 mL)/H_2O (5 mL), and H_2O_2 (30%, 0.43 g, 3.80 mmol) was added. The mixture was stirred at room temperature for 1 h. Then the solid of NaOH (0.38 g, 9.52 mmol) was added and the mixture was stirred at room temperature for 12 h. Then the solvent was removed, and the residue was extracted with MeOH (15 mL), concentrated and dried to give the product 0.72 g with 90.03% yield. ^{1}H NMR (400 MHz, D_2O) δ 8.38 (d, J = 4.8 Hz, 1H), 7.87 – 7.71 (m, 2H), 7.66 – 7.52 (m, 2H), 7.43 – 7.26 (m, 4H). ^{31}P NMR (162 MHz, D_2O) δ 19.42 (s). ESI-MS: [M] calcd for $C_{11}H_9NNaO_2P$, 241.03; found [M+H] 242.20.

1.3 Synthesis of sodium dipyridinyl phosphinate (Nadpp). A solution of 2.5 N n-butyl-lithium (6.40 mL, 16.00 mmol) in 15 mL of Et_2O was cooled to -90 ℃ and 2-bromopyridine (2.52 g, 15.94 mmol) in Et_2O (6 mL) at -90 ℃ was added quickly and the dark red solution stirred at -90 ^oC for 4 h. A solution of phosphorus trichloride (0.74 g, 5.40 mmol) in Et_2O (10 mL) was added dropwise during 1 h at -90 ℃ and the solution stirred at -90 ℃ for 2 h before warming slowly to room temperature. The tan-coloured mixture was extracted with H_2SO_4 (20 mL, 2N) and the extract made alkaline with saturated NaOH solution. The solid product was collected and recrystallized from acetone-petroleum (1:1, v/v) to get pure product 0.59g with 41.09% yield.

Then the product (0.59 g, 2.23 mmol) was dissolved in DMSO (4 mL)/H_2O (4 mL), and H_2O_2 (30%, 0.3 g, 2.67 mmol) was added. The mixture was stirred at room temperature for 1 h. The solid of NaOH (0.27 g, 6.69 mmol) was added and the mixture was stirred at room temperature for 12 h. Then the solvent was removed, and the residue was extracted with MeOH (15 mL), concentrated and dried to give the product 0.51 g with 90.04% yield. ^{1}H NMR (400 MHz, D_2O) δ 8.40 (d, *J* = 4.8 Hz, 2H), 7.91 (t, *J* = 6.7 Hz, 2H), 7.89 – 7.82 (m, 2H), 7.43 – 7.34 (m, 2H). ^{31}P NMR (162 MHz, D_2O) δ 15.70 (s). ESI-MS: [M] calcd for $C_{10}H_8N_2NaO_2P$, 242.02; found [M+Na] 264.95.

1.4 Synthesis of (dfppy)$_2$Ir(ppp). [(dfppy)$_2$Ir(μ-Cl)]$_2$ (1.15 g, 0.95 mmol) and 2.5 equivalent Nappp (0.6 g, 2.37 mmol) were dissolved in 10 mL of 2-EtOCH$_2$CH$_2$OH. After degassed, the reaction was maintained at 135 ℃ for 24 h under argon. Then the solvent was removed and the crude compound purified by column chromatography with $CHCl_3$: MeOH = 20 :1 as eluent. Further purification was taken by gradient sublimation with a yield of 30.01% (0.45 g). ^{1}H NMR (400 MHz, $CDCl_3$) δ 9.67 (d, *J* = 5.7 Hz, 1H), 8.37 (d, *J* = 8.6 Hz, 1H), 8.23 (d, *J* = 8.3 Hz, 1H), 7.94 – 7.86 (m, 1H), 7.78 (ddd, *J* = 10.1, 6.8, 4.2 Hz, 4H), 7.35 (ddd, *J* = 11.8, 7.7, 2.9 Hz, 5H), 7.25 – 7.20 (m, 1H), 7.15 (td, *J* = 7.6, 3.2 Hz, 2H), 6.83 – 6.77 (m, 1H), 6.53 – 6.37 (m, 2H), 5.77 (dd, *J* = 8.8, 2.3 Hz, 1H), 5.61 (dd, *J* = 8.7, 2.3 Hz, 1H). ^{31}P NMR (162 MHz, $CDCl_3$) δ 34.31 (s). ESI-MS: [M] calcd for $C_{33}H_{21}F_4IrN_3O_2P$, 791.09; found [M+H] 792.00. HRMS (ESI-TOF) calcd for $C_{33}H_{22}F_4IrN_3O_2P$ $[M+H]^+$ 792.1015, found 792.0996.

1.5 Synthesis of (dfppy)$_2$Ir(dpp). [(dfppy)$_2$Ir(μ-Cl)]$_2$ (0.96 g, 0.80 mmol) and 2.5 equivalent Nappp (0.5 g, 1.98 mmol) were dissolved in 10 mL of 2-EtOEtOH. After degassed, the reaction was maintained at 135 ℃ for 24 h under argon. Then the solvent was removed and the crude compound purified by column chromatography with $CHCl_3$: MeOH = 15 :1 as eluent. Further purification was taken by gradient sublimation with a yield of 35.08% (0.53 g). ^{1}H NMR (400 MHz, $CDCl_3$) δ 9.53 (d, *J* = 5.0 Hz, 1H), 8.41 (d, *J* = 4.7 Hz, 1H), 8.25 – 8.16 (m, 3H), 7.88 – 7.82 (m, 1H), 7.79 (dd, *J* = 11.2, 4.5 Hz, 1H), 7.75 (d, *J* = 5.6 Hz, 1H), 7.61 (dd, *J* = 12.0, 4.6 Hz, 1H), 7.53 (dd, *J* = 14.7, 6.6 Hz, 2H), 7.41 (dd, *J* = 4.2, 1.6 Hz, 1H), 7.36 – 7.31 (m, 1H), 7.30 – 7.25 (m, 1H), 7.11 (ddd, *J* = 6.8, 3.2, 1.5 Hz, 1H), 6.65 – 6.59 (m, 1H), 6.51 – 6.35 (m, 2H), 5.69 (dd, *J* = 8.8, 2.3 Hz, 1H), 5.58 (dd, *J* = 8.7, 2.3 Hz, 1H). ^{31}P NMR (162 MHz, $CDCl_3$) δ 32.73 (s). ESI-MS: [M] calcd for $C_{32}H_{20}F_4IrN_4O_2P$, 792.09; found [M+H] 793.00. HRMS (ESI-TOF) calcd

for $C_{32}H_{21}F_4IrN_4O_2P$ $[M+H]^+$ 793.0967, found 793.0991.

2. Supplementary data

2.1 Table S1. Crystallographic data for complex (dfppy)$_2$Ir(ppp).

	(dfppy)$_2$Ir(ppp)	Selected Bond	Bond Length (Å)	Selected Angle	Bond Angle (º)
Formula	$C_{33}H_{20}F_4IrN_3O_2P$	C(11)-Ir(1)	2.019(8)	C(12)-Ir(1)-N(2)	95.3(4)
FW	789.69	C(12)-Ir(1)	1.965(8)	C(12)-Ir(1)-C(11)	91.5(3)
T (K)	296(2)	Ir(1)-N(2)	2.007(8)	N(2)-Ir(1)-C(11)	81.8(4)
Wavelength (Å)	0.71073	Ir(1)-N(1)	2.043(8)	C(12)-Ir(1)-N(1)	81.0(4)
Cryst syst	Monoclinic	Ir(1)-O(2)	2.169(6)	N(2)-Ir(1)-N(1)	174.3(3)
Space group	P2$_1$/c	Ir(1)-N(2)	2.007(8)	C(11)-Ir(1)-N(1)	93.9(4)
a (Å)	14.542(16)	Ir(1)-N(3)	2.176(7)	C(12)-Ir(1)-O(2)	174.4(3)
b (Å)	13.256(14)			N(2)-Ir(1)-O(2)	90.1(3)
c (Å)	16.511(18)			C(11)-Ir(1)-O(2)	90.8(3)
α (deg)	90			N(1)-Ir(1)-O(2)	93.8(3)
β (deg)	99.868(18)			C(12)-Ir(1)-N(3)	96.6(3)
γ (deg)	99.868(18)			N(2)-Ir(1)-N(3)	97.2(3)
V (Å^3)	3136(6)			C(11)-Ir(1)-N(3)	171.9(3)
Z	4			N(1)-Ir(1)-N(3)	87.6(3)
ρ_{calcd} (g/cm^3)	1.673			O(2)-Ir(1)-N(3)	81.1(2)
μ (Mo Kα) (mm^{-1})	4.367				
F (000)	1532				
Range of transm factors	1.982 to 25.500				
Reflns collected	17475				
Unique	5822				
GOF on F^2	1.126				
R_1^a,wR_2^b (I>2σ(I))	0.0512, 0.1597				
R_1^a,wR_2^b(all data)	0.0633, 0.1667				
CCDC No.	1439293				

$R_1^a = \Sigma||F_o| - |F_c||/\Sigma F_o|$. $wR_2^b = [\Sigma w(F_o^2 - F_c^2)^2/\Sigma w(F_o^2)]^{1/2}$

2.2

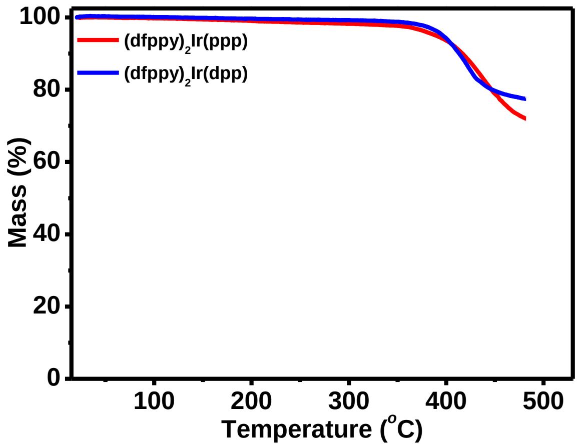

Figure S1. Thermogravimetric analysis (TGA) of $(dfppy)_2Ir(ppp)$ and $(dfppy)_2Ir(dpp)$.

2.3

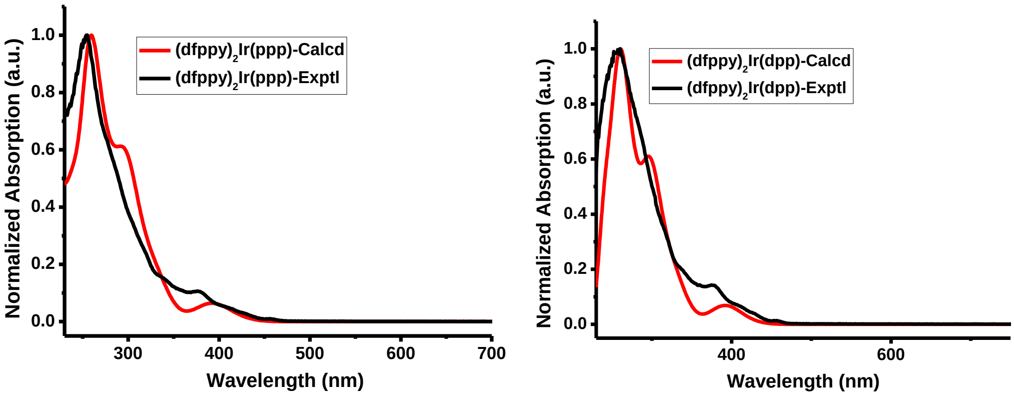

Figure S2. The calculated and experimental measured absorption spectra of the $(dfppy)_2Ir(ppp)$ and $(dfppy)_2Ir(dpp)$ complexes.

Table S2. Orbital distributions of the $(dfppy)_2Ir(ppp)$ and $(dfppy)_2Ir(dpp)$ complexes.

	Orbital Excitations	Transition	Character	Oscillation Strength	Calcd (nm)	Exptl (nm)
$(dfppy)_2Ir(ppp)$	HOMO→LUMO	MLCT	d(Ir)→π*	0.044	394	379
$(dfppy)_2Ir(ppp)$	H-6→L+1	LCT	π→π*	0.0074	261	256
$(dfppy)_2Ir(dpp)$	HOMO→LUMO	MLCT	d(Ir)→π*	0.0503	392	377
$(dfppy)_2Ir(dpp)$	H-6→L+1	LCT	π→π*	0.0079	262	256

2.4

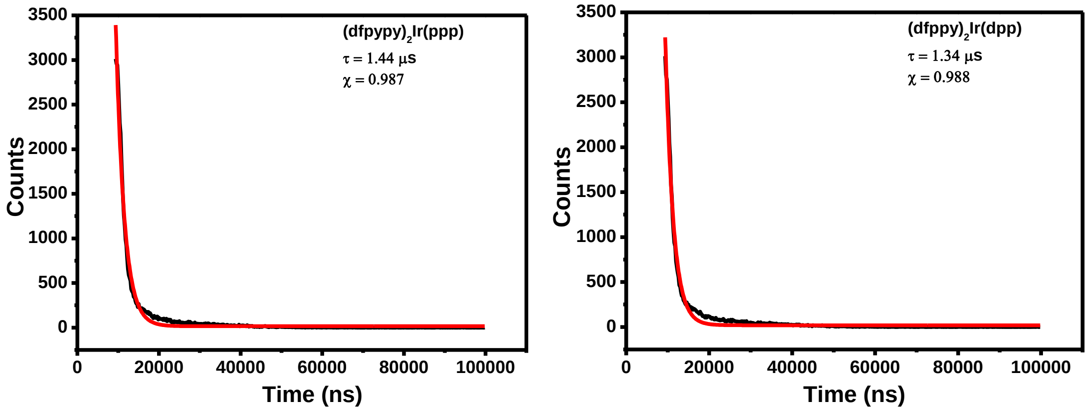

Figure S3. The lifetime curves of (dfppy)$_2$Ir(ppp) and (dfppy)$_2$Ir(dpp) in degassed solution at room temperature.

2.5 Table S3. The relevant data values for HOMO/LUMO levels of (dfppy)$_2$Ir(ppp) and (dfppy)$_2$Ir(dpp).

Complexes	$\Delta E_{Ferrocene}$ [V]	$\Delta E_{complexes}$ [V]	HOMO [eV]	λ_{ab} [nm]	E_g [eV]	LUMO [eV]
(dfppy)$_2$Ir(ppp)	0.133	1.284	-5.95	420	2.95	-3.00
(dfppy)$_2$Ir(dpp)	0.133	1.293	-5.96	412	3.01	-2.95

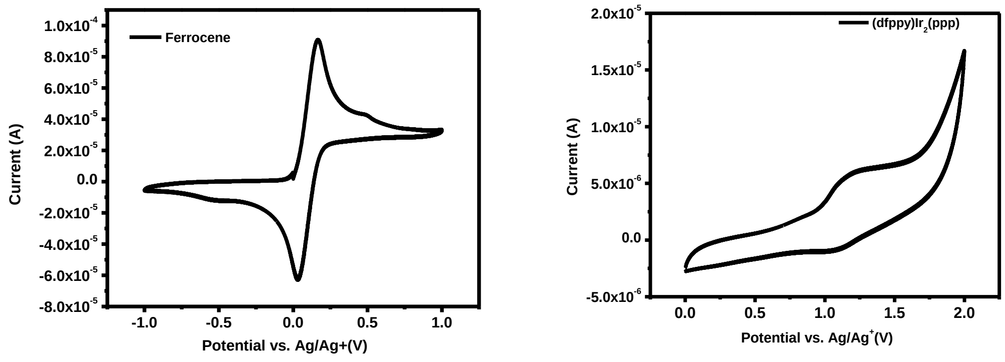

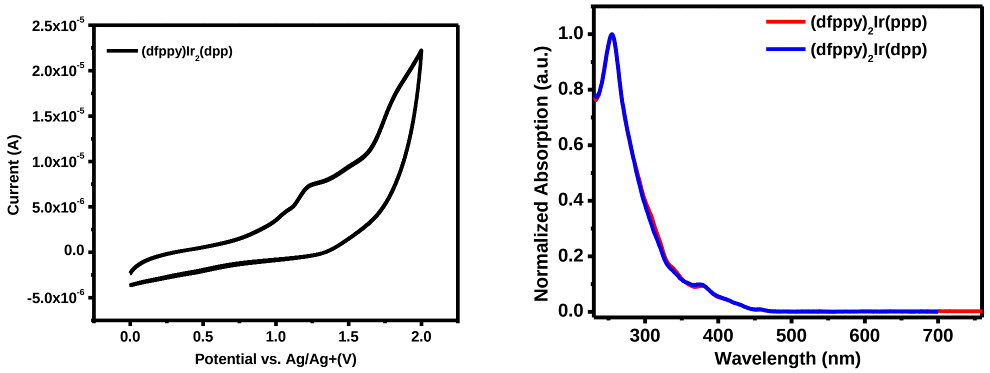

Figure S4. Cyclovoltammety (CV) diagrams of $(dfppy)_2Ir(ppp)$ and $(dfppy)_2Ir(dpp)$ in acetonitrile. CV of ferrocene is shown for the calibration. The absorption spectra of $(dfppy)_2Ir(ppp)$ and $(dfppy)_2Ir(dpp)$ for E_g.

2.6

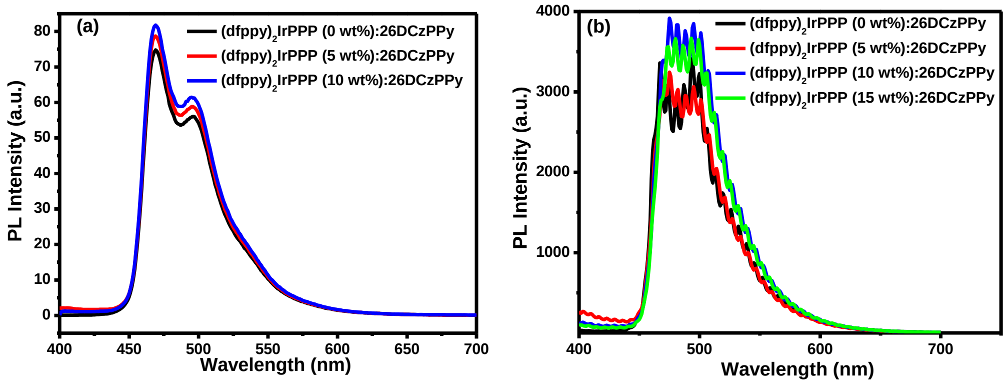

Figure S5. The PL spectra of the $(dfppy)_2Ir(ppp)$ dopant in 26DCzPPy host in (a) CH_2Cl_2 solution and (b) solid film.

2.7 Table S4. Optimization of the doping concentration of Ir(III) emitters.

Device	$V_{turn-on}$ [a] [V]	L_{max}(voltage) [b] [cd/m^2(V)]	$\eta_{c.max}$ [c] [cd/A]	$\eta_{c,100/1000}$ [d] [cd/A]	$\eta_{p.max}$ [e] [lm/W]	$\eta_{p,100/1000}$ [f] [lm/W (V)]
PPP-8 wt%	2.9	34153(10.0)	50.89	49.61(3.5)/ 46.09(4.4)	50.99	44.51/33.89
PPP-1(10 wt%)	2.9	32923(8.9)	52.49	49.71(3.7)/ 46.81(4.6)	51.50	42.19/32.66
PPP-12 wt%	3.0	25944(9.3)	50.80	48.11(3.8)/ 45.38(4.7)	49.85	39.89/31.20
DPP-8 wt%	3.2	26843(9.8)	51.44	51.44(4.1)/ 48.01(4.9)	41.64	39.39/30.76
DPP-1(10 wt%)	3.5	24628(9.2)	55.79	52.79(4.1)/ 48.06(4.9)	50.56	40.20/30.80
DPP-12 wt%	3.5	26730(10.1)	51.21	50.36(4.3)/ 47.08(5.4)	40.44	36.77/28.15

[a] $V_{turn-on}$: turn-on voltage recorded at a brightness of 1 cd/m^2; [b] L_{max}: maximum luminance; [c] η_c: maximum current efficiency; [d] current efficiencies measured at brightness of 100 cd/m^2 and 1000 cd/m^2; [e] η_p: maximum power efficiency; [f] power efficiencies measured at brightness of 100 cd/m^2 and 1000 cd/m^2.

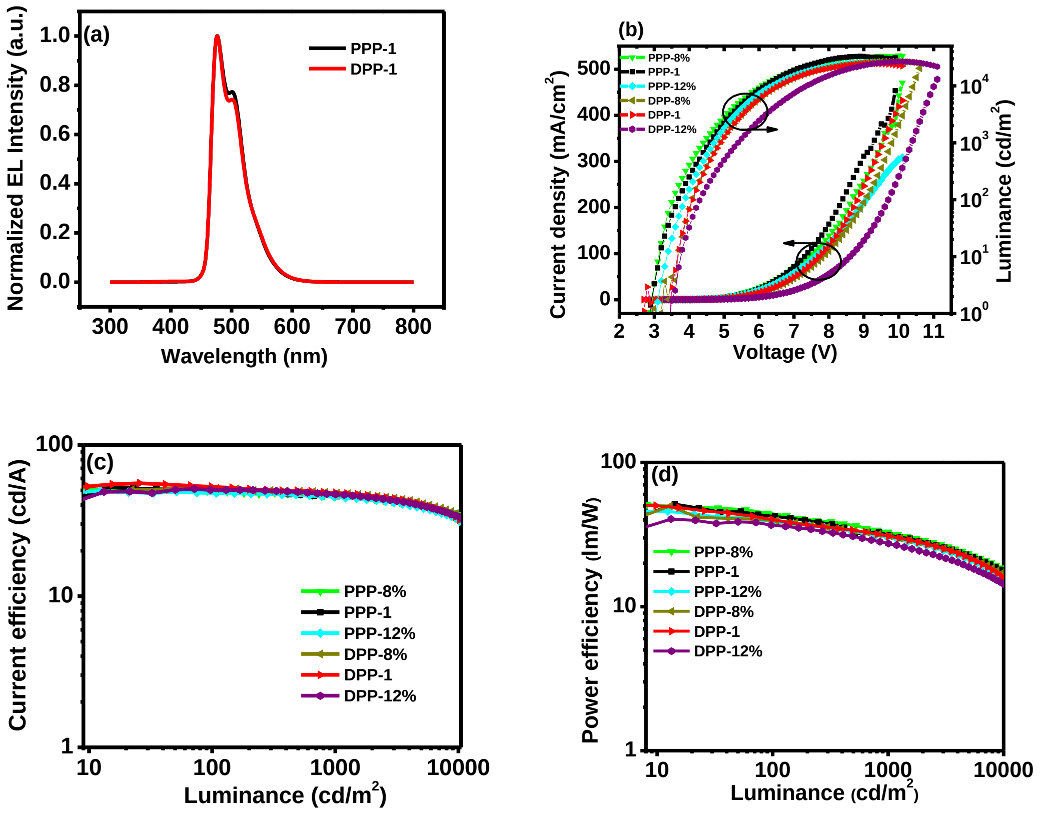

Figure S6. OLED performances with the structure of ITO/MoO$_3$ (3 nm)/TAPC (50 nm)/(dfppy)$_2$Ir(ppp) or (dfppy)$_2$Ir(dpp) (*x* wt%) : 26DCzPPy (15 nm)/TmPyPB (50 nm)/LiF (1 nm)/Al (100 nm). (a) EL spectra at 10 mA. (b) current density and luminance versus voltage. (c) current efficiency as a function of luminance. (d) power efficiency as a function of luminance.

2.8

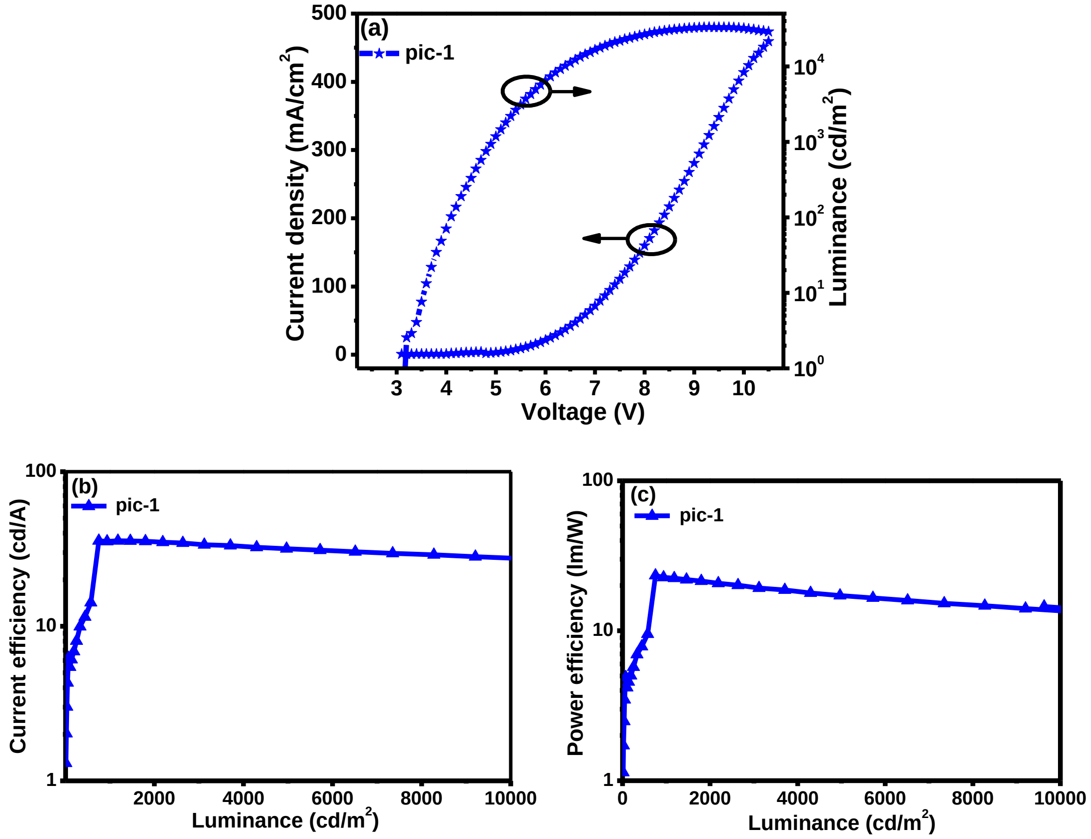

Figure S7. OLED performances with the structure of ITO/MoO_3 (3 nm)/TAPC (50 nm)/FIrpic (16 wt%) : 26DCzPPy (15 nm)/TmPyPB (50 nm)/LiF (1 nm)/Al (100 nm). (a) current density and luminance versus voltage. (b) current efficiency as a function of luminance. (c) power efficiency as a function of luminance.

2.9

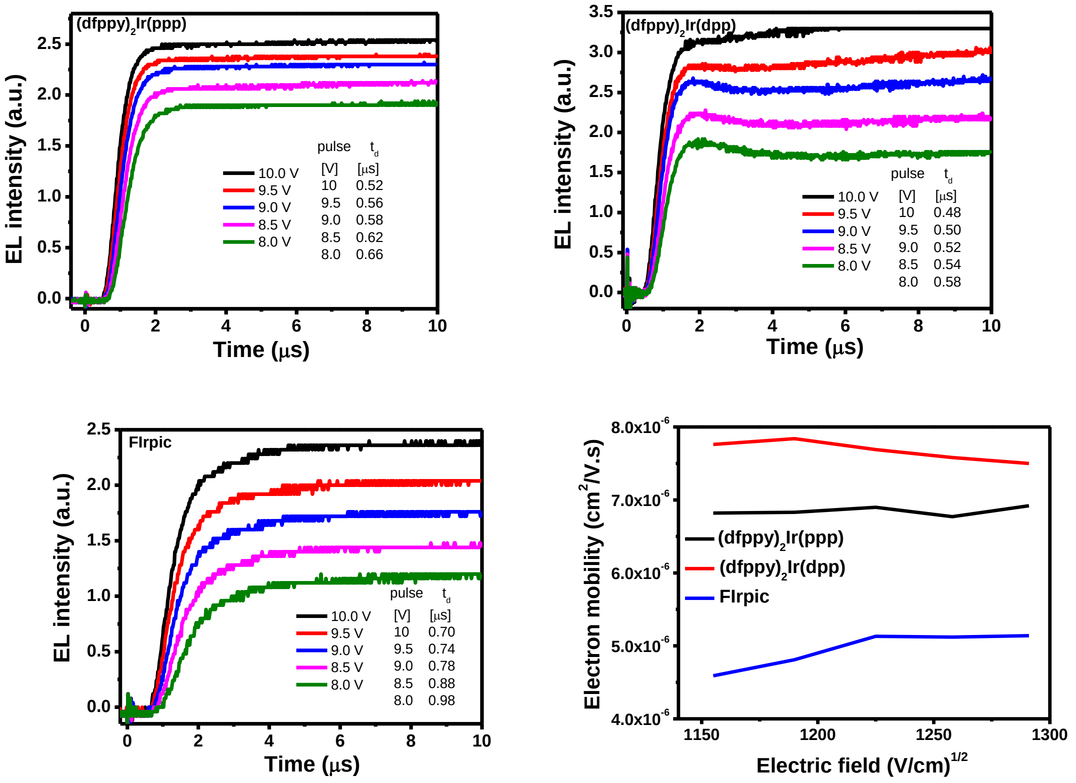

Figure S7. The transient EL signals for the device structure of ITO/TAPC (50 nm)/(dfppy)$_2$Ir(ppp) or (dfppy)$_2$Ir(dpp) (60 nm) /LiF (1 nm)/Al (100 nm) and electric field dependence of charge electron mobility in the thin films of (dfppy)$_2$Ir(ppp), (dfppy)$_2$Ir(dpp) and FIrpic.
